# The Consequences of High Cigarette Excise Taxes for Low-Income Smokers

**DOI:** 10.1371/journal.pone.0043838

**Published:** 2012-09-12

**Authors:** Matthew C. Farrelly, James M. Nonnemaker, Kimberly A. Watson

**Affiliations:** Research Triangle Institute (RTI) International, Research Triangle Park, North Carolina, United States of America; The University of Auckland, New Zealand

## Abstract

**Background:**

To illustrate the burden of high cigarette excise taxes on low-income smokers.

**Methodology/Principal Findings:**

Using data from the New York and national Adult Tobacco Surveys from 2010–2011, we estimated how smoking prevalence, daily cigarette consumption, and share of annual income spent on cigarettes vary by annual income (less than $30,000; $30,000–$59,999; and more than $60,000). The 2010–2011 sample includes 7,536 adults and 1,294 smokers from New York and 3,777 adults and 748 smokers nationally. Overall, smoking prevalence is lower in New York (16.1%) than nationally (22.2%) and is strongly associated with income in New York and nationally (*P*<.001). Smoking prevalence ranges from 12.2% to 33.7% nationally and from 10.1% to 24.3% from the highest to lowest income group. In 2010–2011, the lowest income group spent 23.6% of annual household income on cigarettes in New York (up from 11.6% in 2003–2004) and 14.2% nationally. Daily cigarette consumption is not related to income.

**Conclusions/Significance:**

Although high cigarette taxes are an effective method for reducing cigarette smoking, they can impose a significant financial burden on low-income smokers.

## Introduction

Raising the price of cigarettes is considered to be one of the most effective interventions to prevent and reduce cigarette use [1], [2]. For every additional $1.00 per pack cigarette excise tax, the price of a pack of cigarettes increases by $1.11 [3]. The resulting price increase provides current smokers an incentive to quit or cut back. Among adults, a 10% increase in the price of cigarettes is associated with a 3% to 5% decline in overall consumption, with approximately half of this decline resulting from smokers quitting [4], [5]. However, more recent studies suggest that the effect of higher prices may be diminishing [6]–[8]. For example, Farrelly and colleagues [7] found that a 10% increase in price was associated with a 0.6% decrease in smoking prevalence among adults overall and a 2.7% decrease among young adults aged 18 to 24. Smokers can minimize the impact of tax increases by switching to lower price discount cigarettes; smoking fewer cigarettes more intensively; and/or seeking out low- or untaxed sources of cigarettes, such as in neighboring states, online, or at Indian reservations [9]–[13].

Smoking prevalence is highest among those with low income, low education, and working-class occupations [14]. Several studies have found that lower income groups are more responsive to increases in cigarette prices/taxes [6], [15]–[19], whereas others have found no differences [8], [20], [21]. However, although lower income smokers may be more responsive, cigarette excise taxes are regressive. That is, lower income smokers spend a disproportionate share of their income on cigarette taxes compared to smokers with greater incomes [17]. Gruber and Koszegi [22] argue, however, that if low-income smokers are sufficiently price responsive, tax increases are not regressive and possibly progressive. In 2008, Colman and Remler [17] estimate that smokers in the lowest income tercile spent 7.7% of their income on cigarette purchases, followed by 3.1% and 1.4% for the middle and highest income terciles, respectively. Since this study, cigarette excise taxes have increased substantially in many states, and now 5 states have taxes over $3.00 per pack [23].

In this study, we used data from 2010–2011 to analyze differences in smoking prevalence and consumption overall and by three income levels nationally and in the state with the highest cigarette excise tax ($4.35), New York. We also illustrate the financial burden of cigarette excise taxes on low-income smokers by calculating the amount spent by smokers on cigarettes annually as a share of household income for 2003–2004 and 2010–2011.

## Methods

### Ethics Statement

We obtained institutional review board (IRB) approval for both surveys from the New York State Health Department and RTI International. Both IRBs approved of oral consent, which was documented in the surveys computer assisted telephone interview program.

### Data

The primary sources of data for our analysis are the New York Adult Tobacco Survey (NY ATS) and a National Adult Tobacco Survey (NATS), both sponsored by and available from the New York State Department of Health. NATS is used by the New York Tobacco Control Program to assess progress in New York compared with the nation as a whole.

Our analysis focuses on three measures: current smoking, cigarettes smoked per day by current smokers, and annual household income. Current smoking is defined as having smoked at least 100 cigarettes in a lifetime and currently smoking on some days or every day. Daily cigarette consumption is based on responses to the question “On the average, about how many cigarettes do you now smoke?” Income is assessed with a series of questions, beginning with the question “Was your annual household income from all sources during [year] more or less than $30,000?” Respondents are then asked a progressive set of questions that assesses whether income is less than $20,000 and then less than $10,000 for those who initially state that their income is less than $30,000. Those with higher incomes are asked if their income is greater than $40,000, $50,000, $60,000, $70,000, $90,000, or $110,000 or more. For the current analysis, we collapsed respondents into three income categories: less than $30,000; $30,000 to $59,999; and $60,000 or more. When calculating the share of income spent on cigarette purchases, we set the income level to the midpoint between possible responses (e.g., $25,000 for less than $30,000 and not less than $20,000).

Our analyses are limited to those with complete data on the income and smoking behavior questions. The 2010–2011 sample includes 7,536 adults and 1,294 smokers from New York and 3,777 adults and 748 smokers nationally. In the New York sample, 1,030 adults were excluded due to missing income information (11.9%) and an additional 65 due to incomplete information on smoking status (0.8%). Nationally, 450 and 33 adults were excluded due to missing information on income (10.5%) and smoking status (0.8%), respectively. The 2003–2004 New York sample used to calculate smokers' share of income spent on cigarettes consists of 2,558 smokers with complete information (8.6% of smokers had missing income data).

### Annual Cigarette Consumption and Purchases

To calculate the percentage of a smoker's annual income that is spent on cigarettes, we needed an accurate estimate of cigarette consumption. On average, smokers underreport the quantity of cigarettes they smoke [24]. Using a similar approach to Warner [24], we generate new estimates of underreporting by comparing self-reported cigarette consumption to total cigarette sales nationally. Although smokers can avoid state taxes by purchasing cigarettes online, in border states, and/or on Indian reservations, national sales are less subject to these biases (e.g., sales lost to one state are captured in the neighboring state). We translated annual cigarette sales into an estimate of daily cigarette consumption by dividing sales by the number of smokers and the number of days in the year.
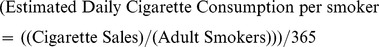
We then compared this to national self-reported daily cigarette consumption.
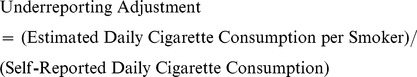
To estimate daily cigarette consumption in New York, we adjusted self-reported daily consumption by the average amount of underreporting nationally.
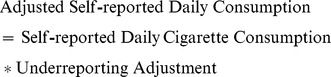
Any difference between adjusted cigarette consumption and sales in New York State represents tax avoidance. To estimate total annual tax avoidance for New York State, we multiplied the daily difference between consumption and sales by 365 days and the number of adult smokers in New York. We then divided this total by 20 to calculate the number of packs and multiplied total packs by the current cigarette excise tax of $4.35 to get total annual lost revenue.

In the NY ATS and NATS, smokers report the price they paid for their last cigarette purchase. Multiplying this price by the smoker's self-reported annual cigarette consumption, adjusted for underreporting, yields an annual expenditure on cigarettes. We then divided this amount by the smoker's annual household income to get an estimate of the share of income spent on cigarettes.
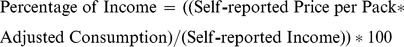
We then summarized this percentage overall and for the three income categories noted above in New York and nationally for the 2010–2011 period. To examine changes in this measure over time, we compared the 2010–2011 period to the 2003–2004 period in New York as the state cigarette excise tax increased from $1.50 to $4.35.

### Statistical Analysis

We begin by reporting descriptive statistics on the prevalence of smoking overall and by income group for New York and the United States. We estimated adjusted Wald tests that account for the complex survey design for overall differences between New York and the United States in smoking prevalence, cigarette consumption, prices paid, and percentage of income spent on cigarettes. To test whether income is associated with smoking prevalence and cigarette consumption while also accounting for the complex survey design and weights, we estimated a logistic regression for smoking prevalence and a linear regression for cigarette consumption.

Given that the relationship between smoking prevalence and income is not necessarily linear, we modeled income with three separate indicator variables, with the lowest income category as the referent. We first estimated stratified models for New York and the United States and performed an adjusted Wald test (a joint test of the significance of the income indicators) to test whether income is related to smoking. We then pooled the data to test whether the relationships between smoking and income differ between New York and the United States. We did this by testing for the significance of interactions between the income indicators and an indicator for New York (i.e., a variable that equals 1 for residents of New York, 0 otherwise). However, properly estimating marginal effects and standard errors for interactions in logistic regressions is complex (Ai and Norton, 2003. For this regression, we estimated a linear probability model which avoids the complexities of estimating and interpreting interaction terms in non-linear models. To verify the results we converted income into a quasi-continuous variable (using income category midpoints) and used methods suggested by Ai and Norton (2003). We then performed linear regressions and repeated the process to test whether the price paid for the most recent pack of cigarettes and the percentage of income spent on cigarettes are related to income level.

Given that household income is missing for 12.7% of the New York sample and 11.3% of the national sample, we imputed which of the 3 income categories those with missing income are predicted to fall within. We did this by estimating a multinomial logit of the 3-level income variable as a function of age, education, race/ethnicity, and gender. We then re-examined whether imputing income influences the relationship between smoking prevalence and consumption and income.

## Results

**Figure 1:**
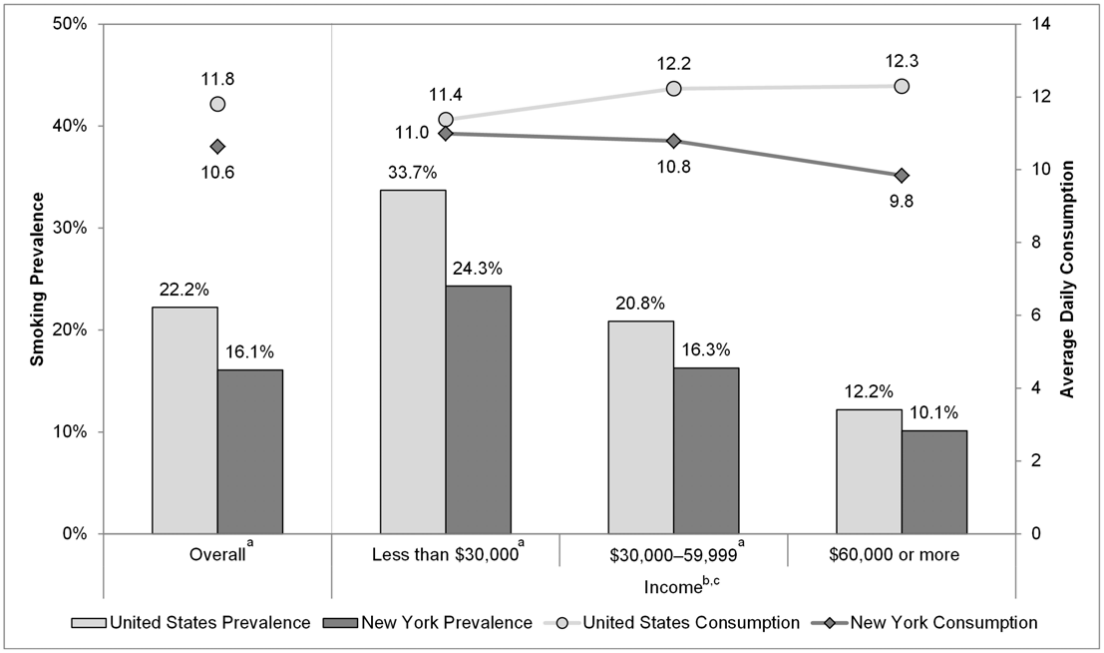
Smoking Prevalence and Consumption Overall and by Income in New York and the United States, 2010–2011. ^a^ Statistically significant difference between smoking prevalence in New York and the United States. ^b^ Statistically significant downward trend in smoking prevalence in New York and the United States. ^c^ As income increases, the prevalence of smoking declines at a more rapid rate in the U.S. compared to in New York State. This figure illustrates that the prevalence of smoking is inversely related to income in New York State and in the United States, with a less pronounced relationship in New York.

The prevalence of smoking in New York (16.1%) is lower than the national rate (22.2%) (*P*<.001) and is strongly related to income in New York (*P*<.001) and nationally (*P*<.001) (Figure 1). Smoking prevalence declines monotonically from 33.7% for the lowest income category to 12.2% for the highest income category nationally. Smoking prevalence is lower in New York than nationally for the lowest (*P*<.01) and middle (*P*<.05) income categories. In New York, there is a similar pattern, ranging from 24.3% to 10.1%. The relationship between smoking prevalence and income is statistically different between New York and the United States in the linear probability model (*P* = .045). The alternative specification that treats income as a continuous variable also shows a statistically significant difference between New York and the United States. Daily cigarette consumption is not related to income in New York (*P* = .517) or nationally (*P* = .730). The statistical significance of these relationships and the patterns illustrated in Figure 1 were not changed after imputing missing income, so we continue to present data only for those with complete data.

Although the average price paid by smokers differs significantly between New York and the United States (*P*<.001), it is not associated with income in New York (*P* = .915) or nationally (*P* = .873). The average price per pack was $7.95 in New York compared with $5.21 nationally.

**Figure 2:**
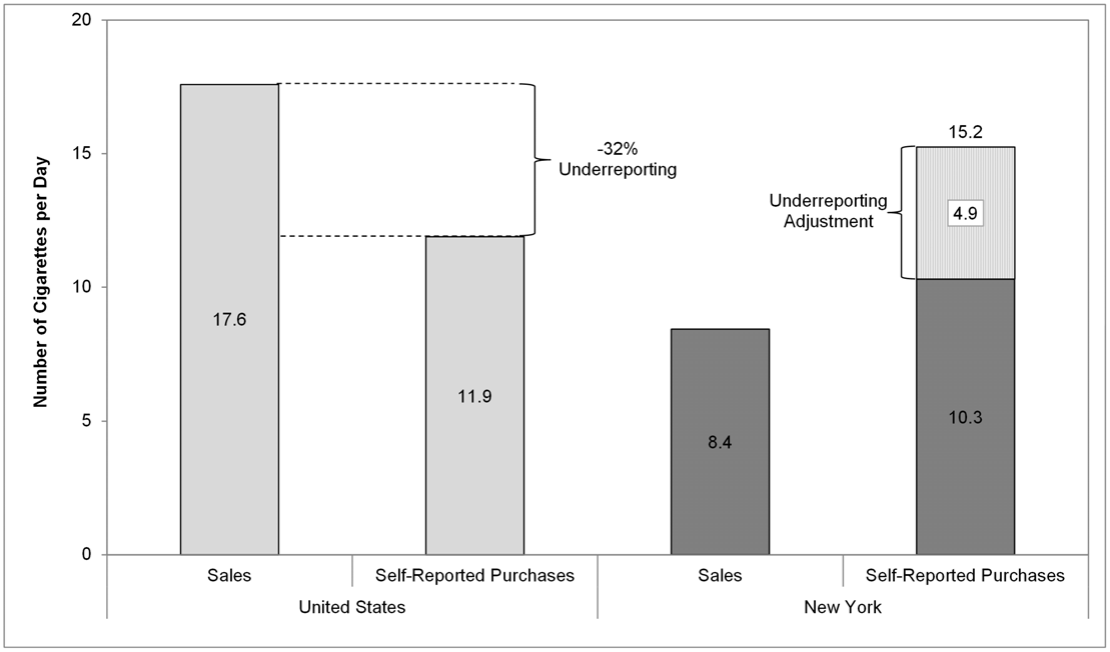
Cigarette Sales and Self-reported Cigarette Consumption per Smoker in New York and United States, 2010. This figure shows that self-reported daily cigarette consumption, adjusted for underreporting, is considerably higher than taxable cigarette sales, suggesting that tax avoidance is significant In New York State.

The comparison between daily sales per smoker in the United States to self-reported daily cigarette consumption indicates that smokers underreport consumption by 32% (17.6 compared to 11.9 cigarettes per day) (Figure 2). This translates to adjusting self-reported cigarettes per day by 1.48 (1/(1−0.32)). Daily sales per smoker in New York are nearly half of what they are nationally (8.4 cigarettes per day). Self-reported daily consumption is 10.3 cigarettes per day before adjusting for underreporting and 15.2 cigarettes per day with the adjustment. In other words, on average, 6.8 cigarettes per smoker per day are purchased outside of New York's tax jurisdiction. This translates to 124 packs per smoker per year or a $541 in lost tax revenue each year for every smoker. Given that the prevalence of smoking in New York is 17.6% in 2010, which translates to 2.65 million smokers, the total lost revenue is $1.4 billion per year.

**Figure 3:**
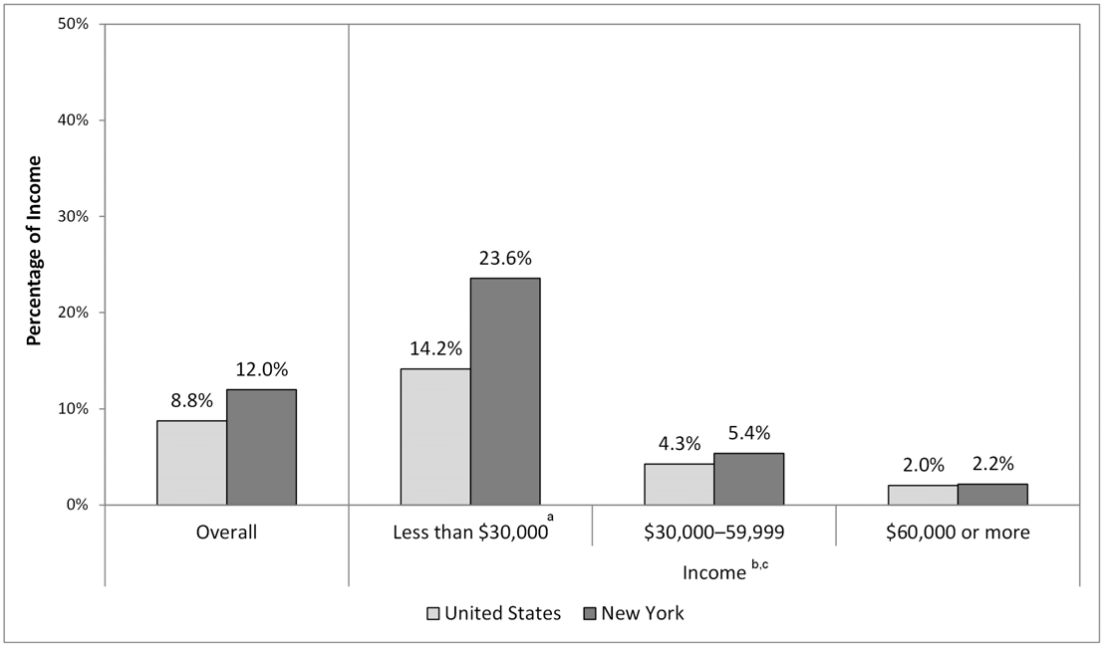
Percentage of Annual Household Income Spent on Cigarettes, Overall and by Income in New York and the United States, 2010–2011. ^a^ Statistically significant difference between the percentage of income spent on cigarettes in New York between 2003–2004 and 2010–2011. ^b^ Statistically significant relationship between income level and the percentage of income spent on cigarettes in New York in 2003–2004. ^c^ Statistically significant relationship between income level and the percentage of income spent on cigarettes in New York in 2010–2011. This figure illustrates that smokers in New York earning less than $30,000 per year spend 21% of their income on cigarettes, compared to 2% for smokers earning $60,000 or more. The comparable percentages for smokers in the United States are 13% and 2% for those earning less than $30,000 and $60,000 or more respectively.

We used the adjusted self-reported cigarette consumption from Figure 2 to calculate annual spending on cigarettes (Figure 3). In 2010–2011, smokers nationally spent 8.8% of their household income on cigarettes, whereas smokers in New York spend 12.0%. This difference varies markedly by income level, especially in New York. New York smokers in the lowest income category spent roughly one-fifth (23.6%) of their household income on cigarettes, compared to 14.2% nationally for smokers with comparable income. The middle-income group spent 5.4% of their income on cigarettes in New York and 4.3% nationally. Smokers in the highest income group spent 2.2% of their income on cigarettes in New York and 2.0% nationally. The relationship between the percentage of income spent on cigarettes and income level differs significantly between New York and the United States (*P*<.05).

**Figure 4:**
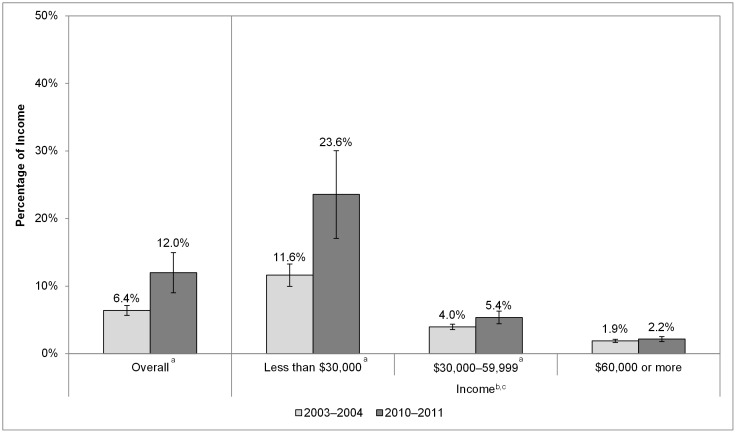
Percentage of Annual Household Income Spent on Cigarettes, Overall and by Income in New York, 2003–2004 to 2010–2011. ^a^ Statistically significant difference between the percentage of income spent on cigarettes in 2003–2004 and 2010–2011. ^b^ Statistically significant downward trend in the percentage of income spent on cigarettes in 2003–2004 and 2010–2011. ^c^ Statistically significant difference between the downward trend in the percentage of income spent on cigarettes in New York and the United States. Between 2003–2004 and 2010–2011, the percentage of smokers incomes spent on cigarettes increased from 6% to 11% overall and from 10% to 21% for smokers with incomes less than $30,000.

Finally, we examined how the percentage of income spent on cigarettes changed in New York over time from 2003–2004 to 2010–2011 as the state cigarette excise tax increased from $1.50 to $4.35 (Figure 4). This analysis illustrates that the percentage of income spent on cigarettes increased over this time period from 6.4% to 12.0% (*P*<.001) for smokers overall and more than doubled for the lowest income category, increasing from 11.6% to 23.6% (*P*<.01). This percentage also increased for the middle-income group from 4.0% to 5.4% (*P*<.01), but not for the highest income group.

## Discussion

The key finding of this study is that cigarette excise taxes impose a significant financial burden on low-income smokers in New York State. Others have also illustrated that cigarette excise taxes have a disproportionate financial burden on low-income smokers [17]. Our national data are similar to Colman and Remler's study with smokers in the lowest income group spending 13% of their income on cigarette purchases, compared to about 8% in 2003 [17]. However, this financial burden is much more pronounced in New York where low-income smokers spend 24% of their annual household income on smoking as result of the high cigarette excise tax. Although we did not have sufficient data to isolate New York City, it is important to note that there is an additional $1.50 tax per pack in New York City. Some have argued [4], [22] that because low-income smokers are more responsive to cigarette price increases than higher income smokers [6], [15]–[19], increases in cigarette taxes may not be regressive. That is, they do not place a disproportionate financial burden on low-income smokers. However, the current study shows that even in the state with the highest cigarette tax, the lowest income group continues to smoke at a much higher rate than the higher income groups.

Recent data suggest that while the prevalence of smoking in New York overall has decreased 20% from 2003–2004 to 2009–2010, those with household incomes less than $25,000 had no statistically significant decline (26.9% to 24.3% based on the Behavioral Risk Factor Surveillance System) [25]. This implies that low-income smokers have not been more price responsive than smokers with higher incomes. In fact, from 2003–2004 to 2010–2011, we find that the percentage of income spent on cigarettes for smokers with annual incomes less than $30,000 more than doubled (11.6% to 23.6%). This suggests that lower income smokers in New York State have not had a greater response to higher taxes than smokers with higher incomes.

Consistent with a considerably higher tax in New York of $4.35 per pack compared with the national average of $1.46 per pack and other tobacco control efforts [26], we also find that smoking prevalence and consumption are lower in New York than in the United States. Smoking prevalence varies considerably by income with a rate for the lowest income group that is more than twice that of the highest income group both in New York and nationally. This is consistent with other studies [14], [17]. This is concerning since disparities in smoking prevalence contribute to the increasing disparity in life expectancies between those in higher and lower socioeconomic groups [27], [28].

We also find that smokers underreport daily cigarette consumption by nearly one-third nationally, similar to Warner's earlier national study that found underreporting of 27% to 36% [24]. After adjusting for underreporting of self-reported cigarette consumption, we find that tax-paid cigarette sales capture only 55% of all cigarettes smoked in New York State. This implies that New York State loses approximately $1.4 billion in revenue as a consequence of tax evasion. Other studies indicate that the primary source of low- or untaxed cigarettes in New York State is from Indian reservations [29]. However, it is important to note that while tax evasion erodes some of the revenue and public health impact of higher cigarette taxes, higher cigarette taxes are still effective in reducing smoking and raising revenue [30], [31], Reducing cigarette tax evasion would exacerbate the regressivity of cigarette taxes in New York State but could also generate significant revenue that could be used to support smoking cessation, especially among low-income smokers.

### Strengths and Limitations

The current study is the first that we are aware of to examine the financial burden of a high cigarette excise tax on low-income smokers. We are also able to contrast smoking behaviors and tax burden between New York State and the United States as a whole. By comparing national self-reported consumption with national sales, we are able to adjust for underreporting of cigarette consumption to better estimate actual daily consumption. However, we cannot be certain whether underreporting is similar across income groups and whether underreporting is different in New York State than the country as a whole. If for example, higher income smokers were more likely to underreport their consumption compared to lower income smokers, then our results would overstate the differences in burden across income groups. However, if the reverse is true, our results would understate the reported differences. However, it is important to note that some lower income smokers have other resources from Medicaid and food stamp programs, so their self-reported earned income does not represent their total resources. In addition, income is missing for a nontrivial number of respondents in both surveys. However, after imputing for missing income, our findings for smoking behaviors by income group were not altered. Our calculations for the share of income spent on cigarettes rely on self-reported cigarette prices and household income, which may both be misreported. Furthermore, if such misreporting varies by income, then our estimates of the differential financial burden by income group could be biased.

### Conclusions

Low-income smokers face a greater financial burden as a result of higher cigarette excise taxes than higher income smokers. Dedicating some of the revenue from cigarette excise taxes for targeted programs that help low-income smokers quit may help alleviate the regressivity of cigarette excise taxes. However, given how the prevalence of smoking has remained stubbornly high among low-income smokers in New York, reducing this disparity will likely prove challenging. To maximize the public health benefits of cigarette excise taxes in New York State, tax evasion needs to be greatly reduced. This would increase the effective price that smokers pay, which would decrease smoking prevalence and daily consumption, while increasing revenue. Unfortunately, this would also likely increase the regressivity of cigarette excise taxes, and thus any efforts to reduce tax evasion should be coupled with additional targeted programs to help low-income smokers quit as well as other programs targeting the poor (e.g., expanded access to health affordable health insurance, food stamp programs, etc.).
